# Using Age-Based Life History Data to Investigate the Life Cycle and Vulnerability of *Octopus cyanea*

**DOI:** 10.1371/journal.pone.0043679

**Published:** 2012-08-17

**Authors:** Jade N. Herwig, Martial Depczynski, John D. Roberts, Jayson M. Semmens, Monica Gagliano, Andrew J. Heyward

**Affiliations:** 1 The Oceans Institute, University of Western Australia, Perth, Western Australia, Australia; 2 Australian Institute of Marine Science, The Oceans Institute, University of Western Australia, Perth, Western Australia, Australia; 3 Centre for Evolutionary Biology, School of Animal Biology, The University of Western Australia, Perth, Western Australia, Australia; 4 Institute for Marine and Antarctic Research, Fisheries, Aquaculture and Coasts Centre, University of Tasmania, Hobart, Tasmania, Australia; 5 Centre for Microscopy, Characterisation and Analysis, The University of Western Australia, Perth, Western Australia, Australia; Leibniz Center for Tropical Marine Ecology, Germany

## Abstract

*Octopus cyanea* is taken as an unregulated, recreationally fished species from the intertidal reefs of Ningaloo, Western Australia. Yet despite its exploitation and importance in many artisanal fisheries throughout the world, little is known about its life history, ecology and vulnerability. We used stylet increment analysis to age a wild *O. cyanea* population for the first time and gonad histology to examine their reproductive characteristics. *O. cyanea* conforms to many cephalopod life history generalisations having rapid, non-asymptotic growth, a short life-span and high levels of mortality. Males were found to mature at much younger ages and sizes than females with reproductive activity concentrated in the spring and summer months. The female dominated sex-ratios in association with female brooding behaviours also suggest that larger conspicuous females may be more prone to capture and suggests that this intertidal octopus population has the potential to be negatively impacted in an unregulated fishery. Size at age and maturity comparisons between our temperate bordering population and lower latitude Tanzanian and Hawaiian populations indicated stark differences in growth rates that correlate with water temperatures. The variability in life history traits between global populations suggests that management of *O. cyanea* populations should be tailored to each unique set of life history characteristics and that stylet increment analysis may provide the integrity needed to accurately assess this.

## Introduction

Effective conservation requires baseline life history data to enable educated decisions to be made for the future management of a species. This includes aspects such as growth, mortality and the reproductive characteristics of an organism. Recently, the implementation of no-take zones based on the timing of life history characteristics, such as peak breeding and brooding periods, has been found to be an effective strategy for the management of artisanal fisheries of octopuses [1]. Age is a particularly important feature because accurate estimates of age provide a context from which to measure other life history characteristics [2], [3], which in turn provides a better understanding of the functioning, lifestyle and vulnerability of the species.

The Day octopus, *Octopus cyanea* [4], is a medium sized, diurnally active octopus that inhabits tropical sub and intertidal reefs of the Indo-West Pacific [5]. While *O. cyanea* is not commercially fished in Australia [6], it is regularly taken off reefs for use as bait by recreational fishermen. At Ningaloo Reef, in remote coastal Western Australia, recreational fishing for finfish is highly regulated. However, the octopus harvest is currently unregulated and the numbers of octopuses caught on this fringing reef remains unknown [7]. While it is difficult to determine the impact from recreational fishermen or provide realistic estimates of densities for such a behaviourally and visually cryptic animal, the biology and life history characteristics of *O. cyanea* provides an insight into their vulnerability as a species and can be accurately quantified.

To date, studies on *O. cyanea* have been limited to a handful of studies on their general ecology, diet, behavioural aspects and oxygen regulation [8]–[14]. However, despite its importance in underpinning their susceptibility to fishing pressure, studies on its life history are limited [8], [15]–[19] and rely on using size to estimate age, a technique which has proven quite inadequate for cephalopods with flexible growth [20].

Stylet increment analysis is a new technique that produces accurate estimates of age that do not rely on the size of the individual [3], [21], [22]. The age of an individual is determined by counting the daily growth increments in the microstructure of the octopus' stylet in much the same way that otoliths are used to accurately age fish. Already validated and documented for the commercially important temperate species, the holobenthic *Octopus pallidus* [3], [21] and merobenthic *Octopus vulgaris* [22], [23], stylet increment analysis has yet to be used to age a tropical octopus species. The aim of this study was to significantly expand on previous research on this common and ubiquitous octopus species by examining the life history characteristics of the intertidal *O. cyanea* population from Ningaloo Reef. Specifically, we set out to (1) develop a reliable ageing technique for a tropical octopus species, (2) provide the first age-based demographic information for the species, and (3) establish what life history stages of O. cyanea are most susceptible to fishing. The results from these findings were used to provide context on their potential vulnerability to recreational fishing pressure at Ningaloo Reef, provide baseline life history information on the species and significantly expand on previous research on this common and ubiquitous octopus species.

## Materials and Methods

### Ethics statement

Research was conducted under the Department of Environment and Conservation Permits #CE002468 and #SF006976 and Department of Fisheries Research Permit #1719-2009-43. University of Western Australia Animal Ethics Committee #F11043, Approval No.RA/3/100/914.

### Collections

Seasonal samples of *O. cyanea* were collected from the same four intertidal reefs within the Ningaloo Marine Park (21°46.626′S, 114°09.975′E to 24°01.865′S, 113°25.235′E), Western Australia in the autumn (April), winter (August), spring (October) and summer of 2009. Individuals were collected by four workers during daylight and crepuscular hours for approximately two hours before and two hours after spring low tides (<0.6m). On each occasion, intertidal reef areas of approximately 0.5–1 km^2^ were thoroughly searched including reef holes, ledges, pools and algal patches at varying distances from the shoreline to ensure good coverage. Most often octopuses revealed themselves through surface water movement resulting from their breathing activities. Upon detection, a few copper sulphate crystals were placed into its den causing it to exit into a hand-held scoop net. From 113 sightings, 102 individuals were captured. Each individual was quickly euthanized by double-sectioning of the brain to sever the optic lobe and brain from surrounding peripheral nerves (as per Boyle [24]), put into a labelled, plastic zip-lock bag and placed into an ice-water slurry. Individuals were weighed, externally sexed by the absence or presence of a ligula on the 3^rd^ right arm (male), and stylets removed by making a vertical cut from the mantle base to the mantle tip on the ventral side. A cut was then made into the mantle at the base of the gills and the stylet removed with a pair of tweezers and immediately stored in vials in a 70% ethanol/freshwater solution before the bodies were immersed in a 5% formalin/seawater solution for future gonad analyses.

### Age, growth and mortality

Each individual was aged using a variation of the stylet increment analysis technique of Doubleday [3] who validated this technique in the holobenthic species *Octopus pallidus* and confirmed the deposition of daily rings, an assumption adopted here for *O. cyanea* [22], [25]. The technique was modified because, in comparison to the temperate *O. pallidus*, the tropical *O. cyanea's* stylets darkened quickly when polished or placed into heated thermo-plastic cement. The modified technique involved cutting a thin transverse section (<1mm thick) from the post-rostral zone of the stylet (as per Doubleday [3]). This section was then simply placed on its end onto a glass microscope slide and covered with a drop of microscope immersion oil to prevent drying out. The position of the stylet was checked under a microscope for orientation and clarity and two direct counts were made by a single reader without reference to the previous count from the centre ring to the outside edge using a hand counter at 200x magnification. A second reader made a single count using the exact same technique at a later date using a new section from the same stylet (original sections had deteriorated) and the means of the three counts used. In cases where sections were not clearly readable along the reading axis (>20% unreadable) or where the closest two counts were not within 10% of each other, a new section of stylet from the same individual was prepared and new counts made. Once counts were completed, the results were compared using the coefficient of variation (CV) for precision. In addition to this, counts were recorded wherever possible where abrupt changes were evident in stylet rings. Abrupt changes in ageing rings such as distinctive darkening of further rings or a change in distance between rings often mark significant stages of an individual's life such as changes from a pelagic to a benthic existence (e.g. [26], [22], [25]).

To examine the growth of *O. cyanea*, a size-at-age plot was constructed using weight and age in days. Weight was the preferred estimate for size as it is traditionally used in cephalopod size measurements because of the plastic nature of using length or width and to enable interspecific comparisons for future studies [27]. Five different growth models (exponential, linear, logarithmic, power and von Bertalanffy) were fitted to the size-at-age data of all individuals and the best model of growth selected based on the highest R^2^ value. To investigate variation in growth rates between (1) sexes and (2) seasons across their lifespan, gender and seasonal data were tested for normality, then examined for homogeneity of slopes using Levene's tests before gender and seasonal regression slopes were statistically compared using ANCOVAs.

Rates of mortality were estimated using Hoenig's equation [28]. Hoenig's equation has been used and found to provide approximate estimates of post-settlement mortality in a host of marine organisms (e.g. [29]) and uses the formula; **ln(Z) = 1.23-0.832 ln(t_max_)** where (Z) is the constant instantaneous rate of mortality and (t_max_) is the maximum age of the species. The maximum age of *O. cyanea* was defined as the oldest octopus collected from the intertidal zone.

### Reproduction

To confirm individual gender and provide information on sex-ratios, size and age at maturity and the reproductive status of individuals among seasons, gonads were histologically sectioned at 6-μm and stained with Gill's Haematoxylin and Eosin. Male and female gonad development was classified into one of four developmental stages following [30], [31]. Using these guidelines, maturity status for each individual was defined as the most advanced stage of gonad developmental maturity and were considered mature upon reaching Stage 3. In males this occurs when spermatids and spermatozoa are abundant and there are no empty spaces between cells and in females when there is a significant increase in oocyte size, deeper infolding of follicular epithelium and increased yolk production [30]. Seasonal sex-ratios and size and age at maturity were examined using logistic regression models. At the population level, age at maturity was defined as the age at which 50% of all individuals of that gender were found to be sexually mature [32]. Seasonal patterns in maturity stage were examined by plotting the frequency of sexual stages for each gender across seasons to provide an indication of the main reproductive season for *O. cyanea*. An ordinal multinomial regression model with a probit link, which allows estimating the effects of more than one independent variable (i.e. sex and season) on a dichotomous dependent variable (i.e. maturity expressed as mature vs immature), was used to examine whether sexual maturity differed between the two sexes over time (i.e. seasons). Coupled with stylet back-calculations of hatching dates, this provides good estimates of peak seasonal trends in reproductive cycles and the approximate seasonal timing of recruitment.

## Results

### Age, growth and mortality

A total of 102 individuals were caught during this study ranging in size from 0.017 – 1.91 kg (mean 0.851 kg ±0.047 kg se). Individuals ranged in age from 48 d to a maximum of 314 d for females and 295 d for males indicating that this species is probably an annual one at Ningaloo, or at best, has a maximum lifespan of approximately 1.5 yrs (e.g. larger animals may reside in subtidal zones). The CV on stylet counts ranged from 0.44 to 11.72% with 90% of the total replicate counts having a CV of less than 10%. This indicated that observer errors were negligible and well within the boundaries of other ageing studies (e.g. [33], [3]).

**Figure 1:**
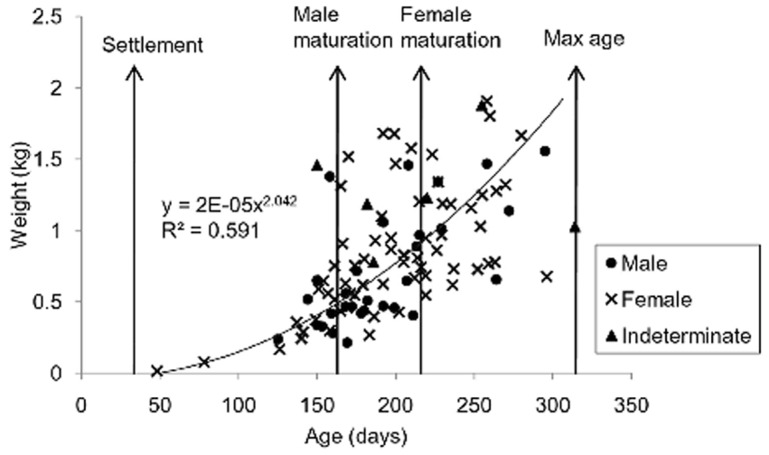
Growth and life cycle of *Octopus cyanea*. Weight at age fitted with a power growth trajectory showing summarised life cycle of *Octopus cyanea* (n = 102) including proposed age at settlement, sexual maturity for each gender and maximum age in days. The 30 d settlement estimate is based on an abrupt change in the spacing of rings bordered by a darker ring in the stylet.

Of the models tested, the growth of *O. cyanea* was best modelled using a power function (Fig. 1, Table 1). Back-calculation of the ages of the 102 specimens indicated a bias towards summer/autumn hatching (28 and 37 individuals respectively) with the remaining 37 individuals hatching in winter and spring (17 and 20 individuals respectively). Comparisons of growth rates between the sexes was determined from 95 individuals (seven octopuses were excluded from the analysis due to gender uncertainty and.data were linearized using a log transform to meet the assumption of normality) and showed no statistical differences (F_1,92_ = 0.38, p = 0.54) allowing male and female growth data to be pooled. Seasonal growth rates (n = 102 individuals) were also not significant (F_3,97_ = 0.87, P = 0.46) indicating that growth did not vary for individuals hatched in different seasons. An abrupt change in the spacing of rings (and a darker ring marking the edge of this) close to the centre of the stylet at around 30 d (+/−7.3 sd, n = 5) was clearly evident in *O. cyanea*. This change most likely marks the transition of the merobenthic *O. cyanea* from a pelagic existence to a benthic reef-associated one (see [22], [25]). Projected rates of daily mortality using empirical estimates were 2.9%.

**Table 1:** Diagnostics and parameters for the five growth models fitted to weight at age data for the Ningaloo *Octopus cyanea* population.

Growth model fitted	R^2^ value	Model equation	Parameter estimates
Exponential	0.470	y = a*e^(bx)^	a = 0.088, b = 0.011
Linear	0.347	y = ax+b	a = 0.006, b = −0.298
Logarithmic	0.336	y = a(ln(x))+b	a = 0.980, b = −4.312
**Power**	**0.592**	**y = ax^b^**	**a = 0.001, b = 2.042**
von Bertalanffy	0.494	y = a(1-e^−bx^)	a = 1.345, b = 0.006

The model of best fit was a power function shown in **bold.**

### Reproduction

**Figure 2:**
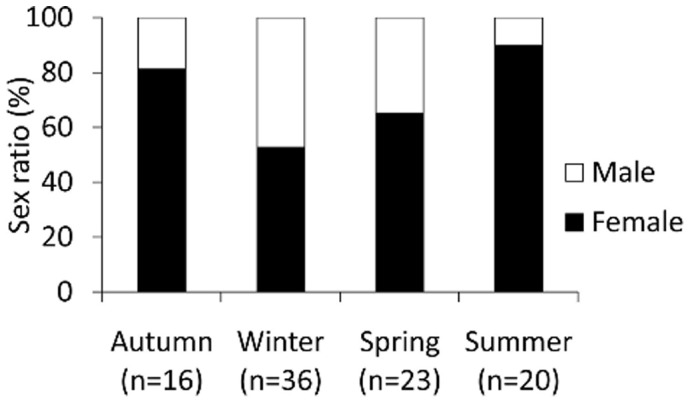
Seasonal gender ratios of *Octopus cyanea*. Seasonal gender ratios in Ningaloo *Octopus cyanea* population.

The sex-ratio of the intertidal *O. cyanea* population was statistically significant with a female skewed ratio of 0.46∶1 (χ^2^ 3.89, df = 1, p = 0.048). However, there was some variation among the four seasons (Fig. 2) with winter samples being equally represented by both sexes (winter χ^2^ 0.00, df = 1, p = 1). For all other seasons, females dominated the population assemblage (autumn χ^2^ 19.78, df = 1, p = 0.000; spring χ^2^ 4.60, df = 1, p = 0.032; summer χ^2^ 38.1, df = 1, p = 0.000).

Size and age at maturity both varied between the sexes (size at maturity χ^2^ 6.24, df = 1, p = 0.013; age at maturity χ^2^ 6.13, df = 1, p = 0.014). Males matured at substantially younger ages and much smaller sizes than females despite the fact that there was no significant difference in growth rates between the sexes. Males were mature at 155 days of age and/or 0.35 kg with the smallest mature male weighing 0.33 kg. In contrast, females matured at around 225 days and/or 1.35 kg with the smallest mature female weighing 0.52 kg.

**Figure 3:**
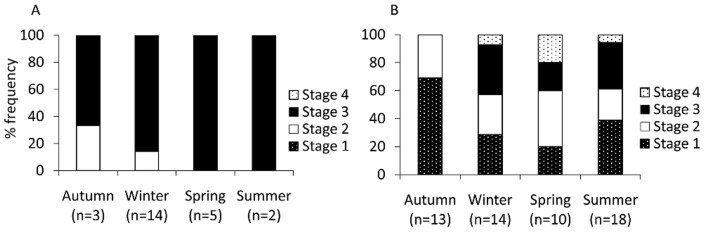
a–b. Seasonal frequency of maturity stages of *Octopus cyanea*. Seasonal frequency of developmental maturity stages of (A) male (B) female *Octopus cyanea*.

Examination of the seasonal frequency of maturity stages indicated a degree of synchrony between the sexes (Figs 3a and b). The ordinal multinomial model indicated there was no difference in the way each sex matures with respect to season (Wald statistic  = 0.28, df  = 1,7, p = 0.60). The most obvious trend for the Ningaloo intertidal population was the total absence of mature (> Stage 3) females coinciding with the lowest rate of male maturity during autumn alongside maximum female maturity coinciding with maximum male maturity in spring and summer. With the exception of autumn, all other seasons exhibited mature individuals of both sexes to some degree with spring and summer appearing to be the peak reproductive season for both sexes. This corresponds well when cross-referenced with the back-calculation hatching dates of specimens biased towards summer and autumn (two thirds of all samples) as well as our 30 day pelagic larval duration estimate. Moreover, mating was directly observed at Ningaloo in early summer. Despite seasonal peaks in reproduction over the spring/summer months, however, it seems likely that staggered reproduction and recruitment takes place throughout most of the year.

## Discussion

Using stylet increment analysis as the basis for exploring the life history of *O. cyanea*, this study expands significantly on previous research and demonstrates the first use of this technique in ageing a tropical octopus species. The intertidal *O. cyanea* population from Ningaloo conforms to many known cephalopod life history generalisations. They appear to be short-lived (<1 yr), displaying variable but rapid non-asymptotic growth coupled with early maturation and the likelihood of high mortality rates. All of these life history characteristics are consistent with documented and theoretical expectations for animals that exhibit short life spans (e.g. prey species, populations under fishing pressure etc) [34]. However, comparisons with other published work revealed marked differences in life history traits between our Ningaloo population and lower latitude tropical populations. This provides a unique and compelling opportunity to investigate the degree of plasticity in life history traits within the species.

The disparities in life history traits between our Ningaloo population and others around the world are large and intriguing suggesting caution in directly comparing stylet ageing studies with the more indirect ageing methods employed in past studies (e.g. laboratory extrapolations, using size to estimate age). Nonetheless, the discrepancies are so large as to be of some interest. For example, maximum ages for Hawaiian *O. cyanea* are 400 d and the largest sized octopuses in Tanzania, Hawaii, and Madagascar are 11.7, 6.5, and 5 kg respectively, contrasting sharply with the 1.9 kg maximum and 314 day lifespan found on Ningaloo's intertidal reefs. One explanation is that because our samples were restricted to intertidal individuals, we simply missed the larger individuals. Relationships between mature female octopus and depth have been well documented [18], [19], [35] and differential size/depth distributions have been reported for other species [36]–38. Another explanation may be that because the timing of life history events is strongly tied to rates of growth and growth rate is strongly dependent on temperature and food availability [16], [20], [39]–[41], then different regions or reefs may produce widely varying life history results in cephalopods. Although we can not comment on food availability, average annual sea surface water temperatures in Tanzania (27.9°C), Hawaii (25.9°C) and Madagascar (28.1°C) [42] are all higher than the Ningaloo average of 24.6°C (Depczynski unpublished data). This suggests that even slight decreases in temperature may adversely affect rates of growth and the timing of life history events for this species. From this viewpoint, it is noteworthy that Ningaloo marks the border between tropical and temperate Australia (Tropic of Capricorn) and represents the southern-most limit for Western Australian *O. cyanea* distributions [6].

The life history characteristics of species evolve in response to their surrounding environment [43]. Species at the limit of their distributions often live in sub-optimal conditions (e.g. thermal limits, paucity of food or habitat resources) and these are often reflected in the life history characteristics of a population [44], [45]. In particular, parameters such as lifespans, growth rates and the timing of maturity can vary considerably across populations [46] with the strongest responses expected in short-lived species with wide geographic ranges [47]. Growth rates and size at first maturity in our intertidal population also contrasted sharply with those in the warmer waters of Tanzania. At a given age, Tanzanian individuals are projected to be considerably heavier than those at Ningaloo or Hawaii. At approximately 6 and 9 months of age for example, Tanzanian octopuses are projected to weigh approximately 0.7 and 4 kg with a female size at first maturity of 0.6 kg [18]. By contrast, Ningaloo females matured as small as 0.5 kg and weighed just 0.5 and 1.3 kg at 6 and 9 months. These weights are more comparable to those found in the Hawaiian population (0.5 and 2.5 kg) where more similar average water temperatures exist (+1.3°C). Despite these apparent slower growth rates, the fact that Ningaloo females still manage to mature at smaller sizes further highlights the geographic plasticity in life history traits for this tropical octopus species and suggests that maturation in the species may be age rather than size-regulated. Moreover, if growth rates vary considerably with temperature, then equatorial populations may have a set of life history traits that support exploitation better than their more temperate cousins.

Alongside natural variability, strong evidence linking shifts in *O. cyanea* population demographics with the intensity of fishing exploitation have also been documented. Guard and Mgaya [18] showed a significant increase in individual mean size and catch per unit effort for one of three Tanzanian sites where fishing was restricted to two rather than five or seven days per spring tide. Despite a two-fold increase in overall fishing intensity (no. fishers/km^2^) at the most restricted site, mean individual weights were approximately double that of the other two sites. Humber et al. [1] reported similar positive effects on catch rates and individual mean size in response to seasonal closures timed to coincide with peak breeding seasons in Madagascar, a strategy recently advocated for *O. cyanea* populations in Mauritius [19]. These increases in size are biologically significant because they shift the average size frequency distributions of populations from one that sits below the minimum size at maturity threshold for females (∼0.5 kg) to well above it. For Ningaloo, larger subtidal individuals probably help ensure the integrity of the *O. cyanea* population. In addition, seasonal resting of the intertidal population during the summer months likely provides some refuge and enables recovery from any fishing-related stress provided recruitment is not largely self-seeding and that outside sources of larvae (including subtidal areas) are not compromised. Recent evidence using DNA parentage analysis in tropical fishes with similar larval durations to *O. cyanea* (∼30 d) has shown that recruitment to natal and nearby reefs makes up a large proportion of reef populations in at least some species [48], [49] although it remains unclear if this is the case for cephalopods.

At Ningaloo, *O. cyanea* are fished from intertidal reefs predominantly for bait via visual hunts on the intertidal reef flats during low tide [7]. While Ningaloo has a small resident human population (<7000), approximately 200,000 tourists visit each year during the cooler autumn – late spring months, a large proportion of whom either fish within the marine park from the shore (48.6% of all visitors) or from a boat (a further 39.7%) [50]. Although the intensity and impact of this fishing on the intertidal *O. cyanea* population remains unclear, some of these fishers collect octopuses for bait from the small number of intertidal reef flats dotted up and down the Ningaloo coast [7]. This recreational fishing activity is largely unregulated (i.e. no size/bag limits or seasonal closures) and legal in most of the Ningaloo Marine Park (NMP) including some sanctuary zone areas near campsites where shore fishing is permitted. Based on the seasonal frequency of maturity stages data presented here, the Ningaloo tourist season begins just prior to and overlaps with the time of highest reproductive activity for intertidal *O. cyanea* (spring). During this period, reproductive activities (e.g. mate searching, courting, mating, egg-laying, brooding etc) increase the already considerable time foraging out in the open across intertidal reef flats (28% – [12]). These activities increase exposure and susceptibility to natural and anthropogenic predators/hunters alike and it is the larger, more conspicuous and mature intertidal individuals that are at greatest risk of detection [18].

### Conclusion

Although this study does not provide representative life history estimates for the entire Ningaloo population, by using age- rather than size-based methods, we provide the first accurate life history estimates for a tropical octopus species including growth, mortality and size and age at maturity. Comparisons with other global locations suggest widely varying life history traits for *O. cyanea* populations although it remains unclear what portion of these differences are attributable to our restricted (intertidal) sampling, different ageing methodologies, or other more natural (e.g. recruitment dynamics, species borders/water temperature) or fishing-related factors. Nonetheless, what is clear is that life history data underpins effective species management strategies and from this point of view, having a reliable age-based technique will vastly improve the life history knowledge base from which management decisions are made. For the Ningaloo population, a number of factors suggest caution should be exercised until gaps in our knowledge can be filled. Information on recruitment dynamics, catch rates, densities and the incorporation of life history data from the subtidal population would provide a proper assessment on the impacts of fishing and further elucidate the degree of variability in *O. cyanea* populations. In the meantime, factors such as the skewed female sex ratios in targeted intertidal areas, peak breeding seasons coinciding with maximum visitor numbers, expectations of increasing numbers of fishers and the current lack of regulations suggest that the precautionary principle should be adopted in the management of this population.
